# Unusually stable abnormal karyotype in a highly aggressive melanoma negative for telomerase activity

**DOI:** 10.1186/1755-8166-1-20

**Published:** 2008-08-22

**Authors:** Sarantis Gagos, George Papaioannou, Maria Chiourea, Sophie Merk-Loretti, Charles-Edward Jefford, Panagiota Mikou, Irmgard Irminger-Finger, Anna Liossi, Jean-Louis Blouin, Sophie Dahoun

**Affiliations:** 1 Division of Genetics, Biomedical Research Foundation of the Academy of Athens, Greece (BRFAA); 2 Department of Gynecology, Laikon Peripheral General University Hospital, Athens, Greece; 3 Genetic Medicine, University Hospitals of Geneva, Geneva, Switzerland; 4 Laboratory of Molecular Gynecology and Obstetrics, Department of Gynecology and Obstetrics, Geneva University Hospitals, Geneva, Switzerland; 5 Department of Cytopathology, Laikon Peripheral General University Hospital, Athens, Greece

## Abstract

Malignant melanomas are characterized by increased karyotypic complexity, extended aneuploidy and heteroploidy. We report a melanoma metastasis to the peritoneal cavity with an exceptionally stable, abnormal pseudodiploid karyotype as verified by G-Banding, subtelomeric, centromeric and quantitative Fluorescence in Situ Hybridization (FISH). Interestingly this tumor had no detectable telomerase activity as indicated by the Telomere Repeat Amplification Protocol. Telomeric Flow-FISH and quantitative telomeric FISH on mitotic preparations showed that malignant cells had relatively short telomeres. Microsatellite instability was ruled out by the allelic pattern of two major mononucleotide repeats. Our data suggest that a combination of melanoma specific genomic imbalances were sufficient and enough for this fatal tumor progression, that was not accompanied by genomic instability, telomerase activity, or the engagement of the alternative recombinatorial telomere lengthening pathway.

## Introduction

Cutaneous malignant melanomas are highly aggressive tumors with unpredictable biological behavior [1]. Metastases in brain, bones and viscera with subsequent ascites development, are frequent [1]. The progression of a transformed melanocyte to malignant melanoma is accompanied by gradual acquisition of multiple genetic alterations that lead to losses of onco-suppressor genes and increased tumor hypermutability [2]. Malignant melanomas display both types of known genomic instability in neoplasia; chromosomal instability (CIN) and microsatellite instability (MIN) [2,3]. MIN has been observed in 30% of cutaneous malignant melanomas [4]. However, the great majority of malignant melanomas examined by various cytogenetic methods, exhibit increased karyotypic complexity, extended aneuploidy and heteroploidy [5-7]. Recurrent chromosomal imbalances in skin melanomas include losses of chromosomes 1p, 6q and 9p [2,8]. Tumor progression and aggressive behavior have been associated with imbalances of chromosomes 7, 10 and 17 [2,7].

Most human tumors including melanomas maintain sufficient telomere length for continuous growth by expressing telomerase [9,10], the remainder are thought to utilize a variety of telomere recombination mechanisms termed alternative lengthening of telomeres (ALT) [11]. Observations on transformed and tumor cell lines that lack telomerase, linked ALT phenotype to highly increased structural chromosomal instability and extreme telomeric length deviation ranging from very long to extremely short telomeres [11]. We report a MIN, CIN, and TRAP negative (Telomere Repeat Amplification Protocol), highly aggressive melanoma metastasis to the peritoneal cavity, with unusually stable abnormal pseudodiploid karyotype, and relatively short but not dysfunctional telomeres.

## Methods

### Immunochemistry-Cytopathology

Cell material from the peritoneal aspirations was subjected to routine diagnostic cytopathology protocols including Giemsa, Papanicolaou, Hematoxylin-Eosin (BDH-Chemicals) stains and immunocytochemistry using the melanocyte specific antibody S-100 (Dako). Immunocytochemical staining against S-100 was performed using Horse Radish Peroxidase (HRP) (Dako). Cell smears were re-hydrated, treated with 3% hydrogen peroxide for 15 minutes and rinsed with Tris Buffered Saline with 0.05% Tween 20 (Dako). After cooling for 20 min, sections were incubated with the primary antibody (rabbit antihuman monoclonal S-100 antibody, 1:400 dilution, Dako) for 1 hour at room temperature and then incubated for 45 min with an anti-mouse HRP labelled polymer (EnVision+System-HRP, Dako). Finally slides were treated with a diaminobenzidine (DAB) chromogenic substrate (Dako) for 10 min, counterstained with hematoxylin, dehydrated and coverslipped.

### Short term cultures/Cytogenetic analysis

Malignant cells from two peritoneal aspirations were collected by centrifugation (10 min/1500 rpm/25°C). They were subsequently cultured in eight 25 cm^2 ^T-flasks at 37°C and 5% CO_2_, in Dulbecco's Minimum Essential Medium supplemented with 10% fetal bovine serum, 0.08 mg/ml amphotericin, 25 units/ml penicillin, and 25 pg/ml of streptomycin (Invitrogen). When high mitotic index was reached, cells were exposed to colcemid (0.1 μg/ml) (Invitrogen) for 30 min, in 37°C, and harvested using trypsine (Invitrogen), after 0.075 KCL hypotonic treatment and Methanol/Acetic acid (BDH-Chemicals) fixation. For the construction of the representative karyotype, we combined G-Banding after Trypsine and Giemsa (GTG-Banding), inverted 4',6-diamidino-2-phenylindole (DAPI)-banding, subtelomeric FISH (TeloVysion-Vysis) and telomeric FISH (Dako), in a total of 400 metaphases from 8 short term monolayer cell cultures. For dual-color interphase or metaphase FISH we used satellite probes specific for chromosomes 7 and 17 (Cytocell). In brief, our general FISH protocol was based on pepsin pre-treatment, formamide or NaOH target denaturation, over-night hybridization and high stringency post hybridization washes. Telomere-specific Peptide Nucleic acid Analog (PNA) hybridizations were performed using a Cy3-(Indocarbocyanine)- conjugated (CCCTAA)_3 _probe (Dako), according to manufacturer's instructions. All FISH preparations were mounted and counterstained with VectaShield antifade medium (Vector), containing 0.1 μg/ml DAPI (Sigma). GTG-Banding was performed after trypsine denaturation (Invitrogen) and Giemsa (BDH-Chemicals) staining. Digital images were captured in a Perceptive Systems Imaging, a Metasystems or an Applied Imaging molecular cytogenetics workstations equipped with fluorescent Zeiss, or Nikon microscopes. Quantification of telomeric PNA fluorescence was performed in 500 chromatids on DAPI counterstained metaphase preparations in a single hybridization experiment using the Isis software (Metasystems).

### Microsatellite instability assay

Two mononucleotide markers, BAT-25 and BAT-26 were tested for microsatellite instability by radioactive PCR after Polyacrylamide Gel Electrophoresis (PCR-PAGE) assay, using the following primers: BAT25.1 (5'-TCGCCTCCAAGAATGTAAGT-3'), BAT25.2 (5'-TCTGCATTTTAACTATGGCTC-3'), BAT26.1 (5'-TGACTACTTTTGACTTCAGCC-3') and BAT26.2 (5'-AACCATTCAACATTTTTAACCC-3'). Experiment was monitored by controls for human microsatellite stability (normal genomic and MIN DNA from a patient with human Hereditary Non-Polyposis Colon Cancer – HNPCC-).

### TRAP assay

Telomerase activity of cell lysates was analyzed by the telomeric repeat amplification protocol (TRAP) assay with a TRAPeze Telomerase Detection kit (Intergen) according to manufacturer's instructions. Approximately 10^6 ^cells were harvested and lysed in 400 μl of 1× CHAPS (3-[(3-Cholamidopropyl)dimethylammonio]propanesulfonic acid, 3-[(3-Cholamidopropyl)-dimethylammonio]-1-propanesulfonate) lysis buffer [Tris-HCl 10 mM, pH 7.5; 1 mM EGTA (ethylene glycol tetraacetic acid), 1 mM MgCl_2_, 0.5% CHAPS 10% glycerol, DEPC (Diethylpyrocarbonate) treated water on ice for 30 min. Cell debris were spun down for 20 minutes at 12,000 r.p.m at 4°C. Each reaction was carried out by using 2 μl of supernatant, 1 μl of each primer, 0.5 μl of Taq-Polymerase (TAKARA), 10 μl of solution-Q (Qiagen), 5 μl of 10× buffer, 2 μl of dNTPs, in DEPC treated water in final volume of 50 μl. The primers used for the TRAP-assay PCR, were TS-5'-AATCCGTCGAGCAGAGTT-3' and Cxa-5'-GTGTAACCCTAACCCTAACCC-3'. The PCR program consisted first of an incubation at 30°C for 30 min and then in a thermocycler, 94°C for 2 min; 94°C for 30 s, 50°C for 25 s, 72°C for 30 s (33×); 72°C for 1 min. PCR products were electrophoresed in a 10% 19:1 acrylamide gel (Sigma)/0.5× TBE (Tris/Borate/EDTA) buffer using the mini protean II gel system (Biorad). Gels were stained with 2 μl of SYBR Green (Sigma) for 15 min at room temperature in 50 ml of TBE 0.5× buffer, and then exposed to UV light and visualized by a Kodak image acquisition station.

### Flow FISH

To measure cellular telomere length, short term cultured cells were hybridized in situ with a fluorescent telomere-specific peptide nucleic acid probe, according to manufacturer's protocol. Briefly, cells were washed in PBS, and re-suspended to 10^5 ^cells/100 μl of a hybridization mixture (Dako) containing 70% formamide and a telomere-specific FITC (Fluorescein isothiocyanate)-conjugated PNA probe. Control samples were re-suspended in hybridization solution without probe to obtain background fluorescence values. After hybridization, cells were spun down and washed twice with 4 ml PBS (Phosphate Buffered Saline) at 40°C for 10 min and finally re-suspended in PBS containing 0.1% Bovine Serum Albumin, 10 μg/ml RNase A (Roche) and 0.1 μg/ml propidium iodide (Calbiochem-Novabiochem). Cells were analyzed on a FACScan flow cytometer (Becton Dickinson) or stored at 4°C before analysis.

## Results

### Patient history and ascitic fluid samples

**Figure 1:**
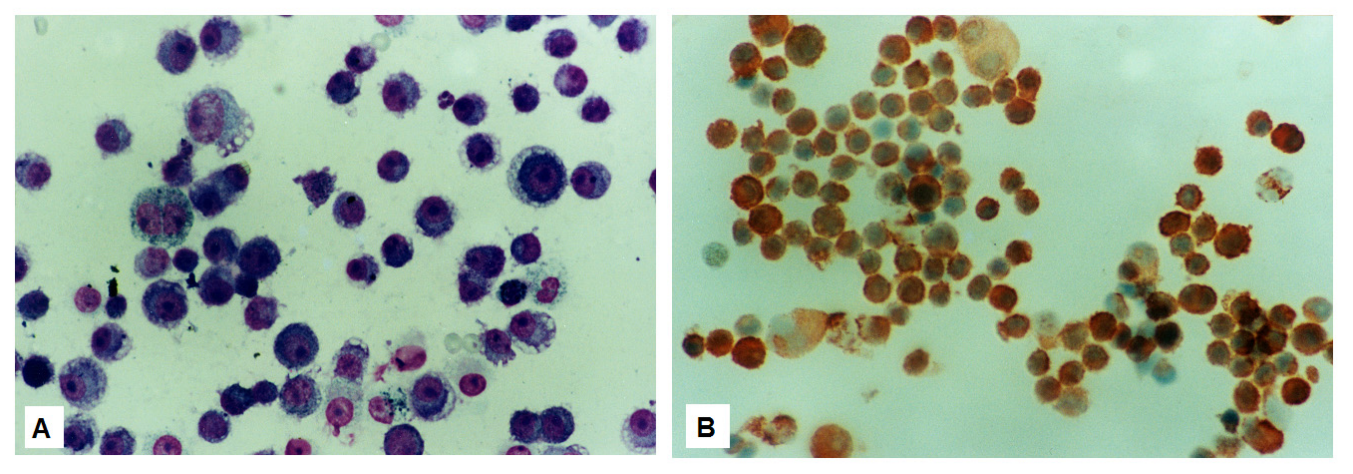
**The cytologic examination of the ascitic fluid showed malignant cells with high mitotic index (Giemsa × 400) (A). **Immunocytochemistry against the melanocyte specific antibody S-100 confirmed the presence of malignant melanocytes (Hematoxylin and DAB × 400).

Peritoneal fluid samples were obtained by two subsequent paracenteses (within a 12-day interval) of a 38-year-old woman, presented at the Department of Gynecology, Laikon Hospital, with ascites and solid structures at her ovaries as revealed by CT-scan. Two years ago the patient had a less than 1.5 cm large, cutaneous nevus excised from the anterior surface of her left hip. The primary tumor was characterized as a nodular melanoma, Clark's level 3, Breslow's depth 2.0 mm. One out of 14 inguinal nodes, excised in a subsequent operation, was found to be invaded. She received 6 cycles of chemotherapy (cis-platin-dacarbazine) and remained disease-free for 15 months. The cytologic examination of the ascitic aspiration confirmed the presence of malignant cells positive for the melanocyte specific antibody S-100 (Figure. 1). The patient refused to be operated, gave her written consent for further research on the specimens obtained, and expired 40 days after presentation.

### Cytogenetic analysis

**Figure 2:**
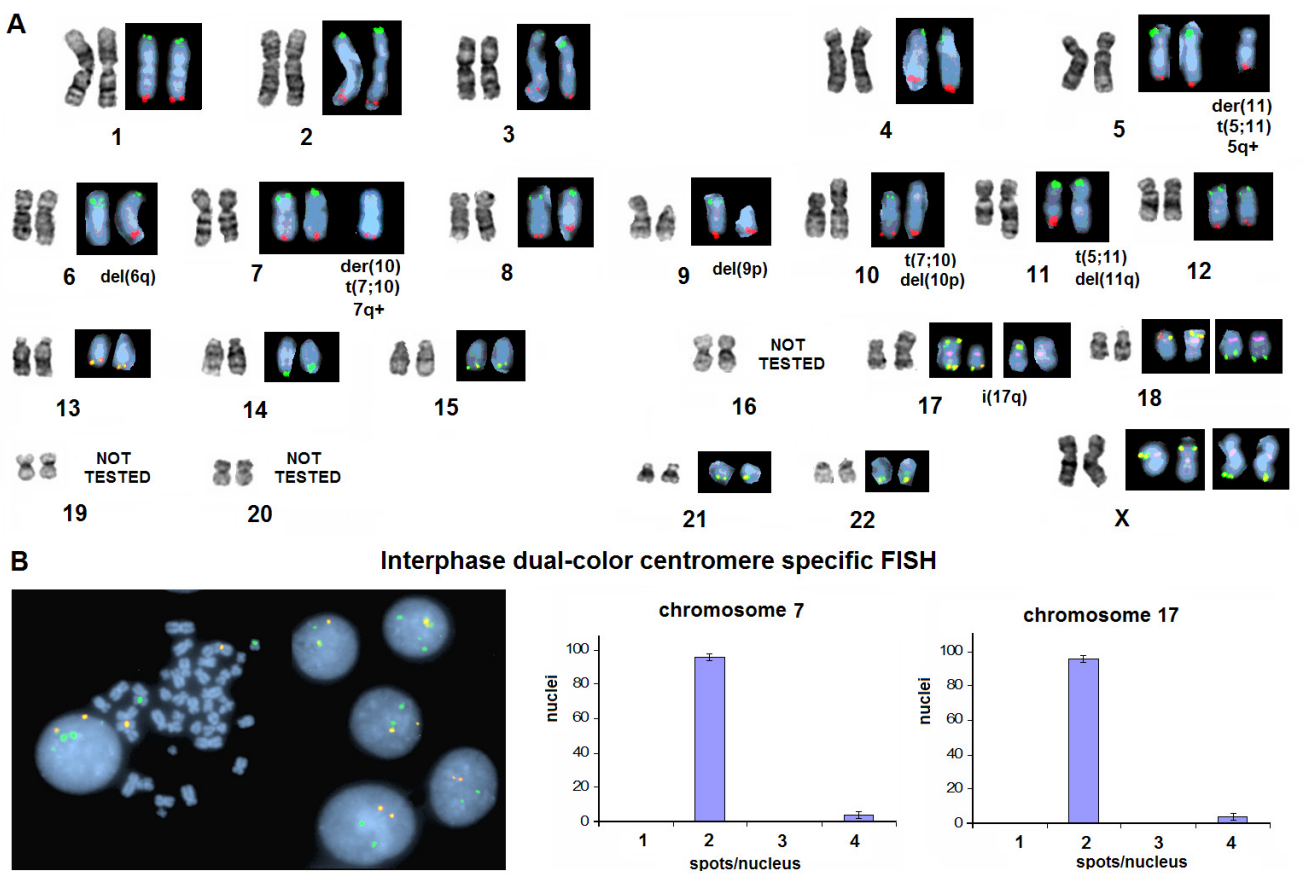
**A GTG-Banding and sub-telomere specific FISH composite representative karyotype of the reported melanoma.** Subtelomeric FISH verified structural integrity of most chromosomes, canonical orientation of both translocations, the deletions 6q, 10p, and 11q, as well as the isochromosome i(17q). The depicted partial dual or triple color subtelomeric FISH karyotypes derive from 23 independent pseudodiploid metaphases; each black box represents a single mitotic nucleus (Red = Spectrum Orange, Green = FITC, Purple = Spectrum Aqua ×1000) (A). Dual color interphase FISH for centromeres 7 (yellow), and 17 (green), shows remarkable numerical stability in 200 nuclei (error-bars represent the standard error of the mean) (B).

G-Banding analysis (according to ISCN 1995) [12] from 8 short-term cell cultures of two peritoneal aspirations taken in an interval of 12 days, showed a 46,XX,del(6)(q23?qter),del(9)(p10pter),der(10)t(7;10)(q31.3qter::p13)del(10)(p14?pter),der(11)t(5;11)(q22.3qter;q23)del(11)(q24?qter),i(17q) pseudodiploid karyotype, in 94–96% of 200 mitoses examined (Figure. 2A). Endoreduplication was observed in 4–6% of the malignant cells leading to a 92,XXXX,idemx2 karyotype. Subtelomeric FISH specific for all human telomeres except for chromosomes 16, 19, 20 and the short arms of acrocentric chromosomes, was used to assist in the description of marker chromosomes identified by G-Banding (Figure. 2A), and to verify deletions spanning up to the end of rearranged chromosomes. To examine if this remarkable karyotypic stability was not confined only to dividing mitotic cells, we performed dual color interphase FISH with probes specific for centromeres 7 and 17, in 200 interphase nuclei obtained from 2 short-term cell cultures from both aspirations. Centromeres 7 and 17 showed notable numerical stability in these populations. The rates of whole genome endoreduplication were similar to those of the karyotyped mitotic cells (Figure. 2B).

### Examination of factors related to chromosome stability

**Figure 3:**
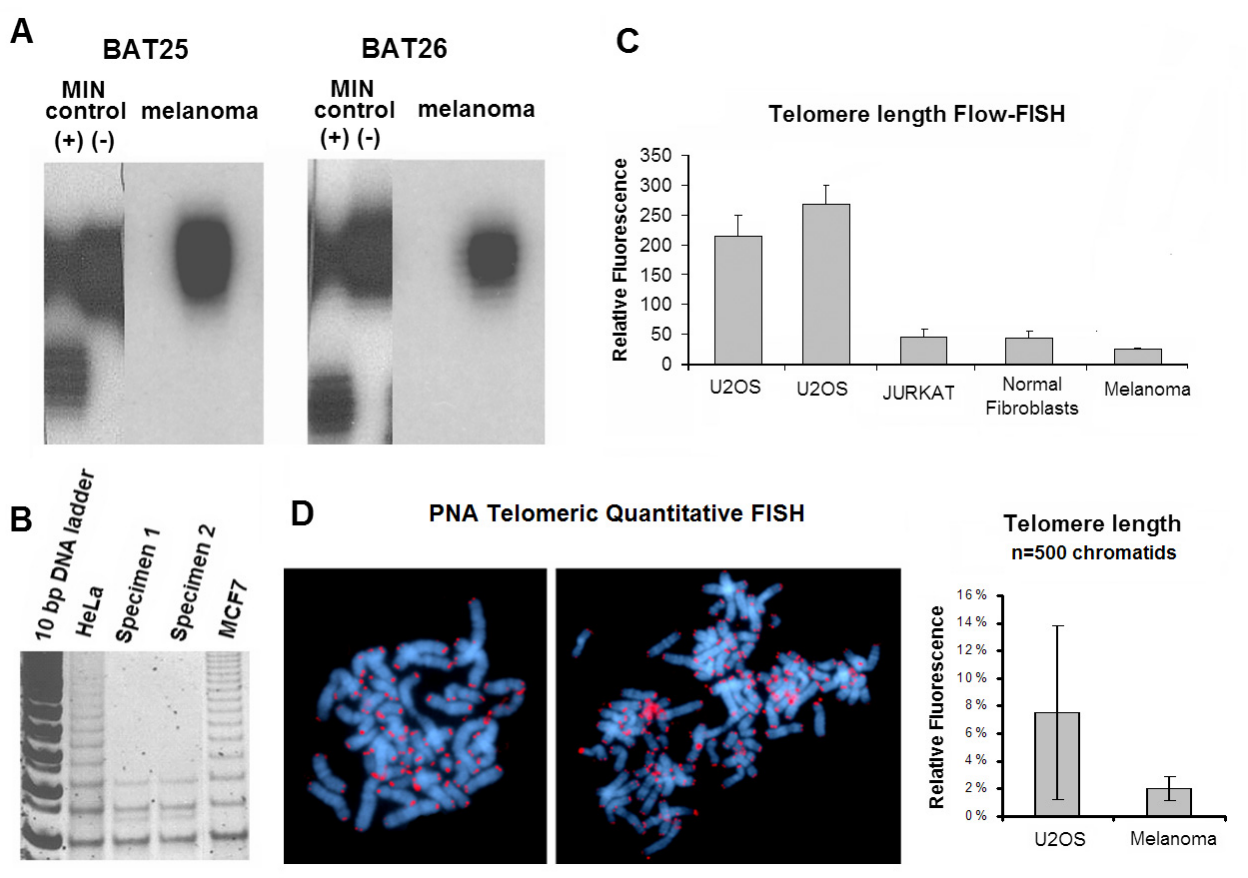
Microsatellite instability in neoplasia (MIN) was excluded in this tumor since the microsatellite markers BAT-25 and BAT-26, showed no instability as compared to MIN positive and negative controls (A). The TRAP assay was negative for telomerase activity in cell culture material obtained from both peritoneal aspirations as compared to two well known telomerase positive human cancer cell lines (MCF-7 and HeLa) (B). Telomere length in this melanoma is relatively low as compared to the ALT U2-OS cell line, leukemic JURKAT cells and human embryonic fibroblasts (error bars represent standard error of the mean between 3 independent experiments) (C). Telomeric PNA FISH indicated uniform terminal capping with TTAGGG repeats on virtually all chromosomes in both pseudodiploid and endoreduplicated clones and low deviation of telomeric length in 500 chromatids as compared to the ALT U2-OS cell line (inverted DAPIx1000) (error bars represent standard deviation)(D).

In an attempt to attribute the karyotypic stability of this metastatic melanoma to measurable parameters related to chromosome stability in the context of neoplastic continuous growth, we examined microsatellite instability (MIN) and telomerase activity. Microsatellite unstable tumors show a significantly lower rate of chromosomal instability as compared to the MIN negative [3]. To rule out underlying microsatellite genomic instability in this metastatic melanoma, we tested by PCR-PAGE the robust mononucleotide repeat markers BAT-25 and BAT-26. Both loci have been shown to be sensitive markers of MIN [13]. Compared to positive and negative controls, this metastatic melanoma displayed no micro-satellite instability (Figure. 3A). Ectopic expression of telomerase in normal fibroblasts has been connected to karyotypic stability [14]. We conducted a TRAP assay to test telomerase activity in cultured cells from 2 sub-cultures from both peritoneal aspirations. In both samples this assay was negative (Figure. 3B). To examine if these melanoma cells followed the ALT-pathway of telomere maintenance [11] we compared the relative telomeric length of our specimen by Quantitative-PNA-Flow-FISH [15] with the pseudodiploid human acute T cell leukemia JURKAT cell line, normal human fibroblasts and an ALT-positive cell line [16]. Cell material for this test was obtained from a short-term subculture that was previously karyotyped and found to be composed exclusively from chromosomally abnormal mitotic cells. This comparison revealed that the melanoma cells had relatively short telomeres (Figure. 3C). PNA-telomeric FISH on 500 chromatids from 10 randomly picked metaphase spreads showed that most of the 46 chromosomes of this metastatic melanoma were uniformly capped with telomeric repeats (Figure. 3D) and no signs of structural chromosome instability attributed to telomere dysfunction such as end-to-end fusions and dicentric chromosomes were evident.

## Discussion

Metastatic transition in most human tumors is accompanied by a series of complex recurrent and stochastic chromosomal anomalies. These changes reflect the evolutionary pressure held by the cancer cells to bypass natural barriers and re-establish continuous growth into unrelated histopathologic environments [17,18]. In this report, the karyotype of the primary tumor is not available, therefore the relative simplicity of genomic imbalances encountered in metastasis, permits only a hypothetical reconstruction of the chromosomal evolution of the disease. It has been proposed that melanomas develop through a mode of karyotypic evolution, common to both low and high complexity karyotypes [2]. To become malignant, an apparently normal melanocyte of this patient underwent multiple karyotypic alterations involving breakpoints in at least 7 different chromosomes as well as chromosomal losses and non-disjunctions. Although we cannot define the temporal order of the recorded rearrangements, we postulate that the hemizygous deletions 6q23qter and 9p- might be early events in the chromosomal evolution of this melanoma. Translocations and deletions involving the q-arm of human chromosome 6 have been found in more than 80% of melanomas [5]. According to Hoglund et al (2005) [19], deletions of the distal 6q should be considered early chromosomal lesions in melanomas. Moreover, the short arm of chromosome 9 is the site of several cell cycle regulators that have been linked with familial disease, or associated to melanoma progression and aggressive behavior [2]. The gains of genomic material and the additional deletions involving 10p, 11q and 17p, were by-products of unbalanced chromatid separation of balanced translocations and the isochromosome formation. These more complex alterations might represent later events in the process of the karyotypic evolution of the disease. Chromosomes 10 and 11 are frequently lost in metastatic melanomas whereas chromosome 7 is frequently gained [2,5,7,20,21].

Rearrangements affecting the short arm of chromosome 17, where the p53 gene is located, have been implicated in the pathogenesis of malignant melanoma [2]. It is interesting that although p53 deficiency has been related to increased rates of numerical chromosome instability or polyploidy [22], in this melanoma hemizygosity of p53 was not associated with continuous genomic instability. MIN tumors display extremely low rates of CIN [3]. We ruled-out the possibility that this melanoma belonged to this type of tumors. We also ruled-out CIN in our specimens, since this metastatic pseudodiploid tumor was highly cytogenetically stable by all means examined. These results are compatible with those of Abdel-Rahman et al. 2001 and Fabarius et al. 2003, who observed that chromosomes of near-diploid cells are structurally much more stable than those of highly aneuploid counterparts [23,24]. Perhaps, the rare, melanoma described here, is unusually stable, because it is near-diploid, in contrast to the majority of highly aneuploid genomically unstable melanomas.

The majority of human malignant melanomas and melanoma cell lines studied with the TRAP assay were found to express telomerase activity [10,25]. Furthermore, telomerase activity has been connected to aggressiveness of melanomas [26]. In continuous neoplastic growth, insufficiently protected telomeres tend to undergo end-to-end fusions and to produce numerous complex chromosome rearrangements such as dicentric chromosomes and inverted duplications [27-29]. No evidence of such lesions was found in our specimens. The transient stage of structural chromosomal instability in this case, equally involved subtelomeric, centromeric and genomic regions, and gave rise to translocations with canonical orientation. Surprisingly, this metastatic tumor was negative for telomerase activity. Moreover, no signs of recombinatorial telomere elongation were present [11] since flow FISH showed relatively short telomeres and PNA FISH displayed a uniform terminal capping of virtually all chromosomes of this melanoma with TTAGGG repeats.

The remarkable stability, and telomeric integrity of the metastatic tumor presented here, can be attributed either to transient telomerase activation, or the action of an unknown but efficient telomere restoration mechanism. However, we can not exclude the possibility that adequate telomeric length for clonal expansion and metastasis was already acquired by the cancer progenitor melanocyte. This assumption might correlate with the relatively young age of the patient. A thorough examination of a series of human osteosarcomas revealed a category of tumors that do not express telomerase activity and do not display any ALT-pathway characteristics [30]. Interestingly these tumors showed low rates of CIN [30]. A similar sub-category might be also encountered in melanomas. The exceptional case reported here, suggests that metastatic progression in this melanoma, was not accompanied by genomic instability, telomerase activity, or the engagement of the classical alternative recombinatorial telomere lengthening (ALT) pathway.

## Competing interests

The authors declare that they have no competing interests.

## Authors' contributions

SG conceived and coordinated the study, carried out the analysis of the results and wrote the manuscript. GP collected the samples, acquired informed consent and took part in the analysis of results and manuscript preparation. MC carried out conventional and FISH cytogenetics. SM-L carried out subtelomeric FISH. C-EJ performed the TRAP and Flow-FISH assays. PM and AL carried out and analyzed the cytopathology assays. II-F participated in the design of the study. J-LB performed and analyzed the microsatellite instability assays. SD participated in coordination of the study and analysis of results. All authors read and approved the final manuscript.
